# A Human-Specific *De Novo* Protein-Coding Gene Associated with Human Brain Functions

**DOI:** 10.1371/journal.pcbi.1000734

**Published:** 2010-03-26

**Authors:** Chuan-Yun Li, Yong Zhang, Zhanbo Wang, Yan Zhang, Chunmei Cao, Ping-Wu Zhang, Shu-Juan Lu, Xiao-Mo Li, Quan Yu, Xiaofeng Zheng, Quan Du, George R. Uhl, Qing-Rong Liu, Liping Wei

**Affiliations:** 1 Center for Bioinformatics, National Laboratory of Protein Engineering and Plant Genetic Engineering, College of Life Sciences, Peking University, Beijing, China; 2 Department of Ecology and Evolution, The University of Chicago, Chicago, Illinois, United States of America; 3 Department of Pathology, Chinese PLA General Hospital, Beijing, China; 4 Institute of Molecular Medicine, Peking University, Beijing, China; 5 Molecular Neurobiology Branch, NIDA, Baltimore, Maryland, United States of America; 6 Department of Biochemistry and Molecular Biology, National Laboratory of Protein Engineering and Plant Genetic Engineering, College of Life Sciences, Peking University, Beijing, China; 7 Behavioral Neuroscience Branch, Intramural Research Program, National Institute on Drug Abuse, NIH/DHHS, Baltimore, Maryland, United States of America; University of California San Diego, United States of America

## Abstract

To understand whether any human-specific new genes may be associated with human brain functions, we computationally screened the genetic vulnerable factors identified through Genome-Wide Association Studies and linkage analyses of nicotine addiction and found one human-specific *de novo* protein-coding gene, *FLJ33706* (alternative gene symbol *C20orf203*). Cross-species analysis revealed interesting evolutionary paths of how this gene had originated from noncoding DNA sequences: insertion of repeat elements especially *Alu* contributed to the formation of the first coding exon and six standard splice junctions on the branch leading to humans and chimpanzees, and two subsequent substitutions in the human lineage escaped two stop codons and created an open reading frame of 194 amino acids. We experimentally verified *FLJ33706*'s mRNA and protein expression in the brain. Real-Time PCR in multiple tissues demonstrated that *FLJ33706* was most abundantly expressed in brain. Human polymorphism data suggested that *FLJ33706* encodes a protein under purifying selection. A specifically designed antibody detected its protein expression across human cortex, cerebellum and midbrain. Immunohistochemistry study in normal human brain cortex revealed the localization of FLJ33706 protein in neurons. Elevated expressions of FLJ33706 were detected in Alzheimer's brain samples, suggesting the role of this novel gene in human-specific pathogenesis of Alzheimer's disease. *FLJ33706* provided the strongest evidence so far that human-specific *de novo* genes can have protein-coding potential and differential protein expression, and be involved in human brain functions.

## Author Summary

For decades, gene duplication, retrotranspositions and gene fusions were believed to be major ways to increase gene number. All involve “mother” genes as the “building blocks” for new genes. However, several recently identified “motherless” genes challenged the idea in that some proteins might have emerged *de novo* from ancestral non-coding DNAs. Did any such genes emerge in human after the divergence from chimpanzee? If yes, such genes might help understand what makes us human. Here we report the first experimentally verified case of a human-specific protein-coding gene, *FLJ33706* (alternative gene symbol *C20orf203*), that originated *de novo* since the divergence of human and chimpanzee. *FLJ33706* was formed by the insertion of repeat elements, especially *Alu* sequences, that contributed to the formation of the first coding exon and six standard splice junctions, followed by two human-specific substitutions that escaped stop codons. The functional protein-coding features of the *FLJ33706* gene are supported by population genetics, transcriptome profiling, Western-blot and immunohistochemistry assays. Data suggest that *FLJ33706* may be involved in nicotine addiction and Alzheimer's disease. *FLJ33706* provided the strongest evidence so far that human-specific *de novo* genes can have protein-coding potential and be involved in human brain functions.

## Introduction

Many mechanisms for the origination of new genes are known, such as tandem gene duplication, retrotransposition, exon shuffling and gene fusion [1]–[5]. By these mechanisms, the origination of new protein coding genes involved “mother” genes that served as blueprints for the new genes. However, recent comparative genomic analysis identified a few “motherless” or *de novo* genes in fly and yeast [6]–[9], which originates from non-coding DNA sequences. It is of great interest to ask whether the human genome also encodes such genes which might contribute to unique human phenotype.

Recently Toll-Riera *et al* identified *in silico* 15 *de novo* human genes which seem to have emerged after the split of primates and rodents [10]. However whether these *de novo* genes encode proteins is unclear due to the lack of protein evidence. More recently Knowles and McLysaght identified *in silico* three human-specific *de novo* genes supported by peptides from high-throughput mass spectrum data [11]. These studies, although tremendously interesting, are lacking in two aspects. First, there is no solid protein evidence so far for any of the *de novo* genes identified—high-throughput mass spectrum data alone as protein evidence can have limitations, as commented by Siepel [12]. Second, none of these genes has been linked to human specific phenotype. Could any *de novo* genes be associated with human unique biology, especially to brain functions?

In our work, we were interested in finding *de novo* genes associated with nicotine addiction. We took advantage of the recently available high-throughput data from genome-wide association studies (GWAS) and data from the more traditional linkage analyses. Unlike candidate gene association studies that usually start with a known gene, GWAS and linkage analyses are hypothesis-free and thus can link previously uncharacterized genes to addiction. Despite the great potentials, current GWAS results are under-analyzed and under-utilized. There is a need for computational protocols to sift through the GWAS results for interesting genes.

## Results

### Identification of *FLJ33706* from GWAS and linkage analyses

Here we carefully re-analyzed results from two published GWAS [13],[14] and two linkage analyses [15],[16] for nicotine addiction and looked for genes that (i) show statistical significance in both GWAS and both linkage analyses; and (ii) have a complete Open Reading Frame (ORF) that has no identifiable homologues in other species. We found an interesting gene, *FLJ33706* (alternative gene symbol *C20orf203*). Both GWAS identified rs17123507, an SNP located in the 3′UTR of *FLJ33706*, as significantly associated with susceptibility to nicotine addiction [13],[14]. Both linkage studies also implicated this region in ‘heavy-smoking quantitative trait’ in individuals of European ancestry [15],[16]. These genetics data established the genomic region of *FLJ33706* as one of the 10 ‘convergent susceptible points’ for nicotine addiction [16]. However, *FLJ33706* was not directly reported as a candidate gene to explain the genetic vulnerabilities in any of the four studies, and to date, *FLJ33706* remains an un-studied gene. In the next steps of our work, we demonstrated that *FLJ33706* is an interesting human-specific *de novo* protein-coding gene. We traced how this fascinating gene originated out of noncoding DNA sequence and experimentally studied its population genetics, mRNA expression, protein expression, and cellular localization.

### Origination of *FLJ33706* out of noncoding DNA

*FLJ33706* is located on Chromosome 20q11.21. Little is known about this gene: it has no publication, no detectable protein domain by InterPro [17], and no BLAST hit to any other known protein sequences. Four mRNAs and four spliced ESTs in GenBank map to this locus, supporting the expression of *FLJ33706* at the transcription level. The UniProtKB/TrEMBL database provided a computationally translated ORF and label it a “predicted protein” (Accession Number: B8JHY2_HUMAN) [18], but the UCSC genome browser and NCBI Entrez Gene database marked it as a “non-coding RNA” [19],[20].

**Figure 1:**
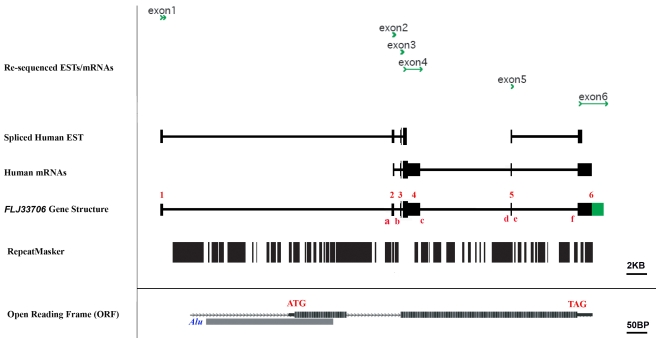
Gene structure of *FLJ33706*, a human-specific *de novo* protein-coding gene. Data for the tracks ‘Spliced Human EST’ and ‘Human mRNA’ was extracted and assembled from UCSC Genome Browser. We re-sequenced all available mRNAs and spliced ESTs, shown in the track ‘Re-sequenced ESTs/mRNAs’. On the basis of these data, we inferred gene structure for this novel gene, with six exons marked as ‘1∼6’ in the track ‘*FLJ33706* Gene Structure’. The exons partially derived from re-sequenced data were highlighted in green. An ORF with two short coding exons located at exon 3 and exon 4 was identified to encode a 194-amino-acid-long peptide (track ‘Open Reading Frame (ORF)’). Newly inserted transposable elements, especially *Alu* sequences, contributed substantially to the formation of the first coding exon and six standard splicing junctions on the branch leading to human and chimpanzee, marked as ‘a∼f’ in the track ‘*FLJ33706* Gene Structure’. All repeat elements in this region were shown in track ‘RepeatMasker’, extracted from UCSC Genome Browser. Coding exons in tracks ‘Spliced Human EST’, ‘Human mRNA’ and ‘*FLJ33706* Gene Structure’ were represented by higher vertical bars, while UTR regions and intronic regions were represented by lower vertical bars. Size scales were added in the figure to give benchmarks for gene sizes. Tracks with different size scales were separated by horizon lines.

We re-sequenced all five available EST clones (see details in Materials and Methods) and inferred the gene structure of *FLJ33706* (GenBank Accession Number: GU931820). The whole locus covers a 42.3 Kb genomic region, encoding a 5,093 bp polyadenylated transcript separated by five standard introns marked with GT-AG splicing junctions. A putative open reading frame (ORF) with 194 codons is located in exons 3 and 4 (**Figure 1**).

**Figure 2:**
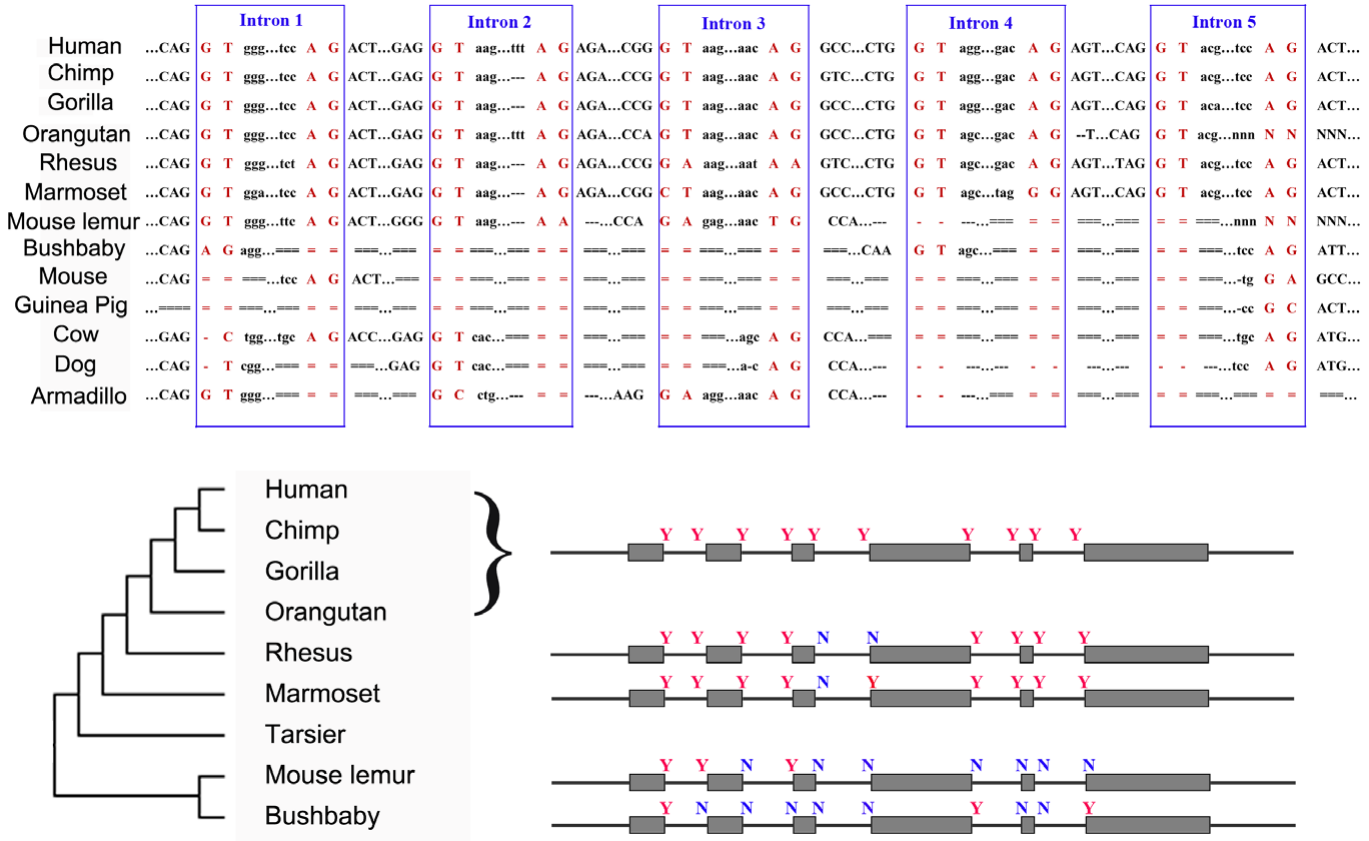
Syntenic alignments of five flanking splicing junctions of *FLJ33706*. (**Top**) The syntenic alignments of five flanking splicing junctions of *FLJ33706* among 13 species were shown, in which intron regions were highlighted in blue boxes and splicing sites were highlighted in red. Lineage-specific insertions, unalignable bases in the gap region and uncertain regions were were marked as ‘-’, ‘ = ’ and ‘N’, respectively. (**Bottom**) The splicing sites from 8 species were shown in the context of the phylogeny. Red ‘Y’ represents presence of the splicing signal and blue ‘N’ represents absence. Exons and introns are not drawn to scale.

The 44-way vertebrate syntenic genome-alignment tracks of the UCSC browser [19] showed that the DNA segment where *FLJ33706* gene is located emerged in the eutherian mammals, since it is completely absent from all outgroups ranging from marsupials to lamprey (**Supplementary Figure S1**). Although this locus predated the radiation of modern mammals, the full splicing structure appeared at a much later time. Specifically, syntenic alignments flanking five splicing junctions (**Figure 2**) revealed that non-primate mammals only encode the first standard splicing junction. For the remaining four introns, non-primate mammals used non-standard junctions if they spliced these regions out at all. Most likely the last four introns were not spliced. Furthermore, only hominoid used GT-AG for the third intron, while the possible ancestral states shared by rhesus monkey and mouse lemur armadillo is GA-AG (**Figure 2**). Such difference across the splicing junctions indicated that the *FLJ33706* locus must have undergone multiple-step changes in order to acquire the present relatively complex gene structure in human.

Manual inspection of the gene structure and vertebrate genome comparisons showed that newly inserted repeat elements, especially *Alu* sequences, contributed substantially to the formation of the first coding exon and the six standard splice junctions on the branch leading to the hominoid (**Figure 1** **,** **2**). Specifically, the splicing acceptor of the second intron, the donor and acceptor of the fourth intron, and the splicing donor of the last intron were derived from *Alu* sequences. In addition, *Alu* contributed to 71% of the first coding exon and 16% of the total ORF. This finding is consistent with other reports that transposable elements can contribute to the creation of both protein-coding regions and splice junctions [10],[21].

**Figure 3:**
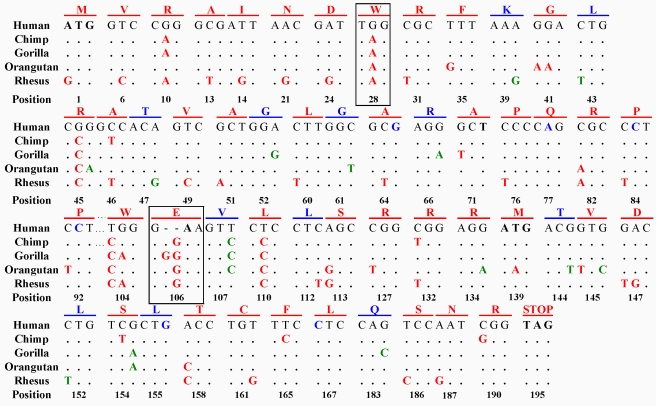
Alignment of human *FLJ33706* ORF with orthologous genomic sequences in four other primates. For each position in human *FLJ33706* ORF, the corresponding orthologous genomic sequences in chimpanzee, gorilla, orangutan and rhesus monkey were aligned to human reference to identify the types of variations. Only amino acid sites with at least one variation among “Human-Human” (SNP), “Human-Chimpanzee”, “Human-Gorilla”, “Human-Orangutan” or “Human-Rhesus” were shown. Identical sites were shown as black dots while divergent sites were shown in red (non-synonymous mutations), green (synonymous mutations) and blue (SNP). Two human-specific mutations that escaped stop codons were highlighted by black frames. Amino acids with non-synonymous variations were highlighted in red while synonymous variations in blue. All sequencing data in this study were traced and manually checked to ensure reliability.

The putative ORF of *FLJ33706* is human-specific. Sequence alignments across multiple primates including human, chimp, gorilla, orangutan, rhesus monkey and marmoset showed that the *FLJ33706* ORF emerged only on the human lineage after the divergence of human and chimpanzee by the introduction of five point mutations, including two important mutations that escaped two ancestral frame-disrupting features, TAG—>TGG at amino acid position 28 and GGAA—>G-AA at amino acid position 106 (**Figure 3**). Chimpanzee seems to share the ancestral status for both of these sites. This is unlikely to be an artifact caused by sequencing error because the sequencing quality of the chimpanzee genome in this region is quite high. For example, TAG is supported by six chimp reads (**Supplementary Figure S2**). Thus, *FLJ33706* is likely a *bona fide* human-specific *de novo* protein-coding gene.

### *FLJ33706* transcription is enriched in brain regions

As aforementioned, eight spliced mRNA and EST sequences support the transcription of *FLJ33706*. These transcripts were mainly cloned from brain libraries, suggesting brain-enriched expression of *FLJ33706*. No mRNA or EST in Genbank from any other species could be reliably mapped to the orthologous genomic locus or to *FLJ33706*—only one unspliced *Sus scrofa* EST (BI343741) could be mapped to the first 3′ untranslated region (UTR) of *FLJ33706*. The GEO [22] microarray database included a databset GSE7094 which profiled five tissues (cortex, fibroblast, pancreas, testis and thymus) in rhesus monkey. Re-analysis of the data showed low expression signal in *Rhesus Macaque* (normalized expression intensity 2.2∼2.7). In summary, both EST and microarray data indicated that *FLJ33706* has low or non-existent transcription in non-hominoid mammals.

**Figure 4:**
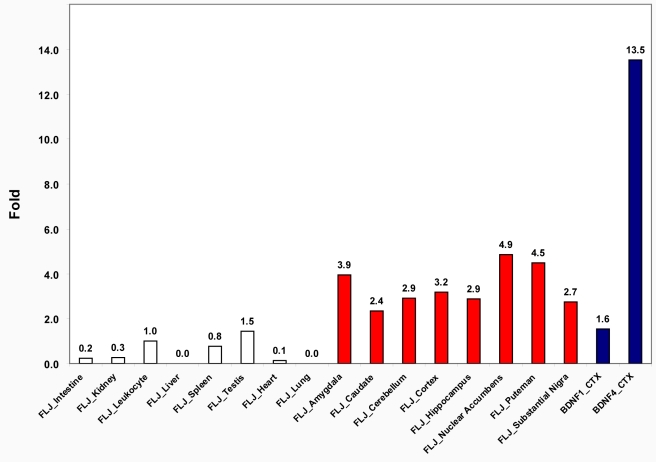
*FLJ33706* mRNA expression in peripheral tissues and brain regions. *FLJ33706* mRNA levels were measured in eight peripheral tissues and eight brain regions using TaqMan-based Real-Time PCR system. Relative quantity was calculated using expression means of human leukocyte as references (Fold = 1.0). *FLJ33706* had relatively higher expression levels in brain regions (highlighted in red) than in peripheral tissues (shown in white). The expression levels of human BDNF1 and BDNF4 (shown in blue bars) in cortex (BDNF1_CTX, BDNF4_CTX) were also compared with those of *FLJ33706* using also leukocyte *FLJ3370*6 expression as a reference.

We further experimentally quantified *FLJ33706* mRNA levels in eight human peripheral tissues and eight human brain regions using the TaqMan technique with FAM-labeled probe hybridized across exon3 and exon4. We observed that *FLJ33706* mRNA was significantly enriched in the brain, especially in regions implicated in cognitive abilities (**Figure 4** **, Supplementary Table S1**). The mRNA expression levels of *FLJ33706* in cortex and hippocampus were comparable to those of the neuronal specific isoform of brain-derived neurotrophic factor (*BDNF1*), although lower than those of the calcium activated isoform (*BDNF4*) [23]. The biased tissue expression patterns of *FLJ33706* and the comparable expression levels between *FLJ33706* and *BDNF1* provided further support that *FLJ33706* might be a functional gene.

### *FLJ33706* encodes a *bona-fide* protein expressed in human brain

To explore whether or not *FLJ33706* may have protein coding potential, we first performed population genetics analysis of 90 individuals including all major sub-populations (**Supplementary Table S2**) to investigate whether this putative coding region, especially the nonsynonymous sites, was under more constraint. We sequenced the coding region and 1 Kb flanking regions of the *FLJ33706* locus in the 90 individuals. No frame-disrupting mutation was found, which suggested some degree of protein-level constraint. Moreover, the nonsynonymous sites showed the strongest constraint (nucleotide diversity *π* of 5×10^−5^) (**Table 1** **, Supplementary Table S2**). By contrast, synonymous sites had an order of magnitude larger *π* (4×10^−4^). We further tested whether this difference departed from neutral assumptions using Hudson's formula [24]. Despite of the small size of this putative protein, the comparison still yielded a marginally significant *p* of 0.1, which suggested that the nonsynonymous sites did evolve under more constraint. Finally, the whole coding region had lower nucleotide diversity *π* compared to its immediate flanking regions, the second intron or the 3′ UTR (**Supplementary Figure S3**). In summary, population genetics analysis suggested that *FLJ33706* potentially encoded a protein under purifying selection.

**Table 1:** population genetics statistics of *FLJ33706*.

Locus	Length (bp)	Number of Single Nucleotide Polymorphisms	*θ*/site	*π*/site
CDS (Whole)	585	4	1.19E-03	1.50E-04
CDS (Synonymous)	151	2	2.29E-03	4.30E-04
CDS (Non-Synonymous)	434	2	8.00E-04	5.00E-05
5′UTR	263	2	1.32E-03	8.00E-05
3′UTR	1,177	8	1.18E-03	4.50E-04
Intron	830	8	1.67E-03	1.00E-03

Population genetics analyses of 90 individuals were performed and population genetics statistics of FLJ33706 were shown. CDS: coding sequences.

**Figure 5:**
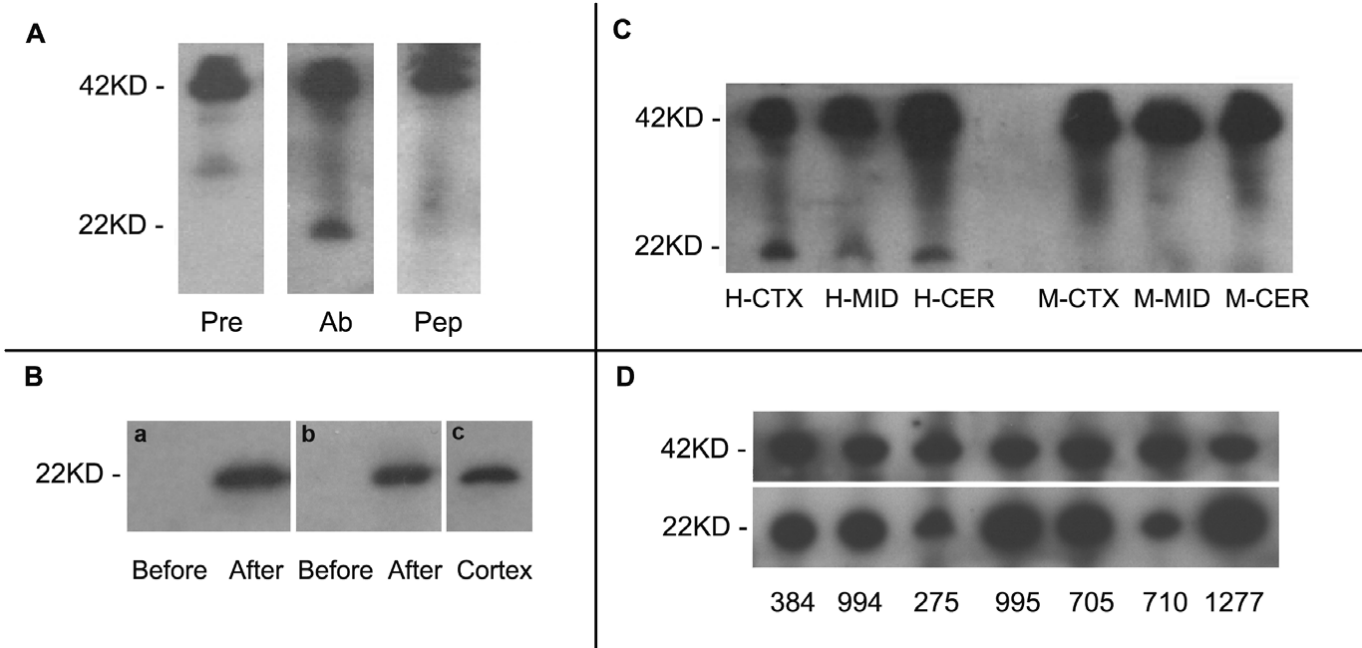
Western-blot assays to determine the protein expression levels of *FLJ33706*. (**A**) A specific band with molecular mass of about 22 kDa in SDS-PAGE, which was consistent with the predicted molecular weight of *FLJ33706* putative proteins, was detected in the Western-blot assay. The band could not be detected in pre-immune control and peptide competition control. Pre: pre-immune reaction assay; Ab: *FLJ33706* antibody assay; Pep: peptide competition assay. (**B**) *E. coli* samples before and after the transformation of FLJ33706 recombination plasmids were assayed by Western blot using (a) His-tag specific antibody and (b) anti-FLJ33706. FLJ33706 expression in human cortex was shown in (c) as the control. Before: *E. coli* samples before the plasmid transformation; After: *E. coli* samples after the plasmid transformation. (**C**) The specific band can be detected in all human brain regions, but not in mouse brain regions used as controls. H-CTX, M-CTX: human/mouse cortex; H-MID, M-MID: human/mouse midbrain; H-CER, M-CER: human/mouse cerebellum; (**D**) FLJ33706 expression can be detected in different human individuals. 384, 994, 275, 995, 705, 710, 1277: individual IDs. 22KD: theoretical molecular weight of FLJ33706 protein; 42KD: molecular weight of beta-actin protein as endogenous control. In A and C, antibodies for FLJ33706 and endogenous control were mixed in Western assays.

However, protein-coding potential of *FLJ33706* suggested by population genetics analysis was still not conclusive. To explore whether or not *FLJ33706* actually encodes the 194-codon protein, we developed FLJ33706-specific antibody and performed Western blot analyses. We designed a 17-amino-acid antigenic peptide, CTSKAQRVHPQPSHQRQ, corresponding to the non-repetitive region (residues 68–83) of the FLJ33706 putative protein plus a cystine at the N-terminus to facilitate conjugation to an adjuvant. The epitope sequence had no homology with the coding peptides of *Alu* or other repeat elements and could not match any other proteins in NCBI NR database [20]. This peptide was synthesized and used to immunize rabbits. The FLJ33706-specific anti-serum was produced from a responsive animal after initial and boosting immunizations. Using this anti-serum as the primary antibody, Western blot assay detected a band with apparent molecular mass of 22 kDa, which was consistent with the predicted molecular weight of the *FLJ33706*-encoded protein, in human brain cortex (**Figure 5A**). This band was not present when pre-immune serum was used or when the antibody was pre-absorbed with excess synthetic FLJ33706 antigenic peptides (**Figure 5A**) [25]. We further expressed FLJ33706 recombination protein with His-Tag in *E. coli* expression strain to evaluate the specificity of FLJ33706 antibody in Western blot assays. As expected, the band with apparent molecular mass of 22 kDa was detected in transformed *E. coli* samples by both His-Tag specific antibody and the aforementioned FLJ33706 antibody, but not in wild-type *E. coli* samples (**Figure 5B**). These results provided verification of the antibody.

**Figure 6:**
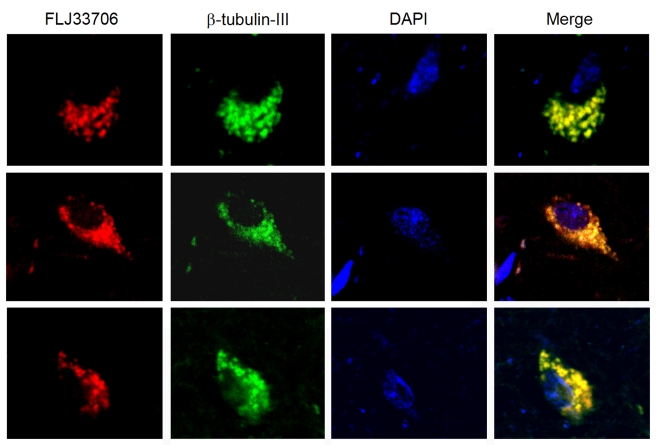
Immunohistochemistry studies of FLJ33706 protein in human brain. Results from confocal immunofluorescence imaging to visualize FLJ33706 protein (red) in normal human cortex were shown. Neurons were marked with beta-tubulin-III (green). The nucleus was also stained with DAPI (blue). The optimal dilution of FLJ33706 antibody was optimised based on the detection of cytoplasmic signal in brain cells. The three rows showed results from three independent experiments.

Using this verified FLJ33706-specific antibody, we studied the expression and localization of FLJ33706. We first identified the expression of FLJ33706 in three human brain regions: cortex, cerebellum and midbrain. The specific band could be detected in all human samples but not mouse samples as negative controls (**Figure 5C**). We then performed within-species studies using cortex samples from seven different human brains and observed FLJ33706 expression in all samples, with some variation in protein expression levels (**Figure 5D**). We further performed immunohistochemistry studies of FLJ33706 by high-resolution confocal imaging in normal human cortex slides stained with beta-tubulin-III. The clear co-localization signals indicated cellular localization of FLJ33706 protein in human neurons (**Figure 6**).

### *FLJ33706* is up-regulated in Alzheimer's disease (AD) brains

Could *FLJ33706* be involved in other human brain-related pathogenesis such as AD? As a preliminary study, we measured the transcriptional level of *FLJ33706* in the middle fontal gyrus (Brodmann area 46) of 20 AD brains and 18 normal brains using the TaqMan-based Real-Time PCR system. The expression level of *FLJ33706* in AD brains was significantly elevated (Mann Whitney Test, *p* = 0.027) (**Supplementary Figure S4**). This finding implicated *FLJ33706* as a potential candidate gene for studying the human-specific pathogenesis underlying Alzheimer's disease [26].

## Discussion

### *FLJ33706* represents a human-specific *de novo* protein-coding gene with the strongest evidence so far

In previous works, only one of the *de novo* genes in yeast and three in human had some high-throughput mass spectrum evidence of protein coding potential [7],[11]. However high-throughput mass spectrum data can be noisy and peptide identification is dependent on the algorithms and search parameters. Our results on FLJ33706 provided the strongest experimental evidence so far of protein expression and differential protein expression of a *de novo* gene.

We experimentally verified the existence of the predicted ORF in human, and observed two frame-disrupting features in chimpanzee that would prevent this ORF from being translated. Moreover, these two features are shared by multiple non-human primates, which suggest that this ORF did not exist in the ancestral status. Identification of ancestral frame-disrupting features is a common strategy to identify species-specific *de novo* proteins [27],[28]. Ideally, we would want to use chimpanzee tissues as negative controls in the Western blot assays. Unfortunately, it proved impossible for us to obtain chimpanzee postmortem samples, especially brain regions, due to our limited resources. Despite this, all our current evidence supports FLJ33706 as a human-specific *de novo* protein.

The recently published genome-wide scan by Knowles and McLysaght identified three human-specific *de novo* protein-coding genes [11] but failed to identify *FLJ33706*. The authors used the automated annotations by Ensembl (version 47) which incorrectly annotated *FLJ33706* as having an orthologous protein-coding gene in chimpanzee (ENSPTRG00000030588). However, as we described before, the chimpanzee locus consists of two frame-disrupting features. In order to make an intact ORF, Ensembl's automatic annotation pipeline made these two features (“TAG” and “G”) as extra tiny introns inside the frame. Such events are extremely unlikely because very few human introns are smaller than 80 bps [17]. In other words, misannotation of Ensembl have likely resulted in the failure of Knowles and McLysaght [11] to discover *FLJ33706*.

Siepel commented on the importance of distinguishing true *de novo* genes from genes that were functional in ancestral genomes but lost in multiple lineages [12]. In the case of *FLJ33706*, the latter scenario is highly unlikely. First, we traced the whole evolutionary history of *FLJ33706* across vertebrates and found that only human has an intact ORF. If this gene were functional in ancestral mammals, then there would have to be too many independent gene loss events, which is highly unlikely. Second, parallel loss for the same locus in different lineages requires that this locus be in some sort of mutational hot spot [12]. Our population survey showed that *FLJ33706* does not have an unusually high level of polymorphism (*θ* ∼0.001 which is comparable to the genome-wide background level of 1×10^−3^) [29]. Thus, at least in human, this locus is not generally permissive for mutation. In summary, *FLJ33706* is a *bona fide de novo* gene.

### *FLJ33706* has the proposed features of *de novo* genes

Siepel proposed a few features of *de novo* genes [12]: *de novo* gene products are usually small with less than 200 amino acids because of the difficulty in *de novo* gene origination; they are often derived from the antisense strand of a pre-existing gene so that they might be able to re-use the transcriptional context; repeats elements might be involved in origination of some *de novo* genes as shown for the gene *hydra* in *D.melanogaster* [8]. *FLJ33706* showed similar features: it encodes a small protein of 194 amino-acids; although it is not derived from the antisense strand of another gene, it is located in a gene-dense region with two other genes in its immediate flanking regions (<30 kb distance) and thus the local chromatin structure might be open, which renders transcription more permissive; and finally, the primate-specific repeat element, *Alu*, contributed to origination of multiple introns and a portion of the coding region.

### Non-neutral evolution of *FLJ33706*

The small protein size and human-specific nature of *FLJ33706* resulted in insufficient statistical power for many evolutionary tests. Nevertheless, we were still able to detect that this locus deviates from neutral expectation. Polymorphism distribution across different functional sites including non-synonymous sites, synonymous sites, UTR and introns suggested that *FLJ33706* is subject to functional constraint. Base-level conservation score calculated by PhyloP [30] based on placental mammal genome alignment showed that introns 2 and 3 are enriched with fast-evolving nucleotides (**Supplementary Figure S5**) which suggested that the emergence of these two introns in primate might be driven by positive selection.

Although this locus existed since at least 80 million years ago (the time for mammalian radiation), its complete splicing structure encoding five standard splicing junctions is younger than 38 million years (human and rhesus monkey divergence time) [www.genome.gov/Pages/Research/Sequencing/SeqProposals/PrimateSEQ012306.pdf]. It is possible that *FLJ33706* is already transcribed in the hominoid ancestor at low abundance. Thus, human FLJ33706 protein may have evolved out of a noncoding RNA which evolved out of noncoding DNA.

Furthermore, *FLJ33706* are mainly expressed in human brain, with more than two folds higher expression in cortex compared to testis. By contrast, its ortholog in rhesus monkey seems to have low expression intensity in major tissues and non-differential abundance between cortex and testis. Thus, *FLJ33706* not only acquired more complicated gene structure, but refined its expression profile in the human lineage.

### *FLJ33706* is involved in human-specific brain pathogenesis

As mentioned above, an addiction-linked SNP rs17123507 is located in the gene region of *FLJ33706*, confirmed by two GWAS and two linkage analyses. To clarify whether this SNP is the ‘causative’ SNP of addiction susceptibility within its haplotype block, we used HapMap data to identify all SNPs showed strong linkage disequilibrium (r2≥0.8) with rs17123507. rs17123507 was the only one located in the exon region (3′UTR) of *FLJ33706* among a tandem set of putative binding sites of *let-7*, a brain-expressed miRNA implicated in neuron specification [31]. All other SNPs were located in intronic or intergenic regions without any annotations or detectable signals of regulatory elements. Thus, rs17123507 was the most possible ‘causative’ SNP within the haplotype block that convey addiction susceptibility.

We also found that *FLJ33706* expressions were up-regulated in AD brains. Thus *FLJ33706* is likely involved in a range of human brain functions and pathogenesis. However, exactly how *FLJ33706* affects human brain functions and exactly why both addiction and AD might be implicated remain unknown and are interesting questions for future studies.

### A model for identifying interesting candidate genes from GWAS data

GWAS provides invaluable links between genes and diseases/phenotypes at high throughput. During the past few years, GWAS have identified numerous genetic variations that contribute to susceptibilities underlying various complex diseases. However, GWAS data is often under-analyzed and poorly interpreted. Our work provides a computational protocol for identifying and studying interesting candidate genes from GWAS of not only addiction, but also other diseases and phenotypes. On the other hand, the studies of the functions of novel genes are time-consuming and often involve much guesswork. Our work demonstrated the feasibility of integrating the rapidly accumulating data from GWAS and linkage analyses to associate novel genes with human diseases and phenotypes.

Our work is a good example of how computational screening of existing biological data can lead to interesting, experimentally verifiable discoveries. Although we spent much effort to experimentally verify the gene and protein expression of *FLJ33706*, the most novel part of our contribution is in fact how we had computationally selected this hidden gem from the human genome in the first place. More specifically, our work can serve as a model for future studies of *de novo* species-specific protein-coding genes that would start from computational and evolutionary analyses similar to what we have done here.

In conclusion, our data provided the strongest evidence so far for a human-specific *de novo* protein and its association with human brain functions. It had been well accepted that protein amino acid changes, protein family expansion and shrinkage, and *cis*-regulatory element changes contributed to human brain evolution [32]. Our study suggested that motherless new genes may be an under-appreciated source of new brain functions.

## Materials and Methods

### Ethics statement

This study was conducted according to the principles expressed in the Declaration of Helsinki. Human tissues were obtained from Department of Pathology, Johns Hopkins Medical School and the NICHD Brain and Tissue Bank, which have been approved by the Institutional Review Board of Johns Hopkins Medical School and University of Maryland, Baltimore, Maryland, USA. All animals were handled in strict accordance with good animal practice as defined by the relevant national and/or local animal welfare bodies, and all animal work was approved.

### Sample preparations

Brain tissues from 20 Alzheimer's disease (AD) patients and 18 non-AD control individuals were obtained post mortem (Department of Pathology, Johns Hopkins Medical Institutions). For each individual sample, a portion of medial frontal gyrus (Brodmann area 46) was prepared for extraction of total RNA. Frontal cortex, midbrain, and cerebellum brain regions were obtained from the NICHD Brain and Tissue Bank for Developmental Disorders at the University of Maryland. Human brain samples used in immunohistochemistry studies were ordered from the Folio Company. The human DNA samples from 90 different individuals were order from Coriell Cell Repositories. Mouse brain samples were prepared in accordance with previous studies [25],[33].

### Re-sequencing and assembling mRNAs and spliced ESTs

Available EST clones for *FLJ33706* (Entrez GeneID: 284805), including BC105014, BG820670, AW196294, H08894 and AI301139, were purchased from Invitrogen CloneRanger™ and sequenced by Invitrogen. Exons of *FLJ33706* were then assembled with Sequencher software (Gene Codes Corporation, USA) using publicly available reliable mRNAs, spliced ESTs and results from our re-sequenced clones.

### RNA isolation, cDNA synthesis, and real-time PCR quantification

RNA isolation, cDNA synthesis, and real-time PCR were performed as described previously [25], using glyceraldehyde-3-phosphate dehydrogenase (Applied Biosystems) as an endogenous control. Brain region and peripheral tissue RNAs were purchased from Clonetech. *FLJ33706* specific Fam-labelled MGB probe across exon 3 and 4 (5′-TGA GCC GGG CCA CAT-3′) and PCR primers (Forward: 5′-TCC CTT TAC AAA AAC TGG AAT GC-3′; and Reverse: 5′-GCA GTG AGT CCA GCC AAG ACT-3′) were designed to detect the transcript. Relative quantity was calculated using expression means of human leukocyte as references. Expression levels of two *BDNF* isoforms in human cortex were used as references, following the protocols proposed in Liu et al [23],[25].

### Population genetics analysis

In order to test the functional constraint of the putative small protein encoded by *FLJ33706*, we sequenced 90 human individuals in different populations (**Supplementary Table S2**). DNA samples were purchased from the Coriell Institute for Medical Research. The *FLJ33706* locus including the coding sequence and 1 Kb flanking regions (intron or untranslated regions) were PCR-amplified using primers designed by Oligo (http://www.oligo.net). When necessary, we ran multiple PCR experiments to amplify the full-length region. PCR bands were sent to Invitrogen for sequencing. For each copy, four walking reactions were performed. Subsequently, we used Phred, Phrap and Consed [34],[35] to assemble the *FLJ33706* locus for each individual. Single nucleotide polymorphisms (SNPs) were identified with Polyphred [36] and Polyscan [37]. Specifically, homozygous or heterozygous SNPs were called by Polyphred first. We retained those highly reliable SNPs with Polyphred score of 99. For SNPs with a score lower than 99, we retained them only if they were also identified by Polyscan. We used DnaSP v4.50 [38] to calculate the statistics of polymorphisms. We calculated the probability of the number of observed segregation sites in nonsynonymous sites on a hypothetical *θ* (e.g. the one in synonymous sites) by following the recursive equations [24]:
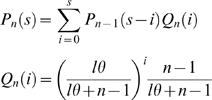



Where, *l*, *n* and *s* are defined as the length of region of interest, the number of alleles and the number of segregation sites, respectively. *Q_n_(i)* indicates the probability that *i* mutations occur when there are *n* ancestral lineages, while *P*_n_(*s*) indicates the probability that *s* sites segregate in a sample of *n* individuals.

### Re-analysis of rhesus monkey microarray data

We found in Affymetrix *Rhesus Macaque* Genome Array a probeset MmugDNA.22336.1.S1 for the orthologous locus of *FLJ33706*. We also found a GEO [22] dataset, GSE7094, which profiled five tissues (cortex, fibroblast, pancreas, testis and thymus) in a rhesus monkey with six replicates for each sample [39]. We downloaded GSE7094 raw array files from NCBI GEO database [22]. We used R and Bioconductor [40] platform to handle this data. Specifically, we used GCRMA [41] to do background subtraction, normalization and probe summarization, and Microarray Suite, version 5.0 (*MAS5*; *Affymetrix*) to call presence or absence.

### Expression of FLJ33706 recombination protein in *E. coli*

We expressed FLJ33706 recombination protein in *E. coli* expression strain. The full-length coding region of FLJ33706 was obtained by PCR amplification using an isolated human genomic library as the template. The PCR products were ligated by T4 DNA ligase and the resulting full-length fragment was sub cloned into the pET-28a expression vector with Poly His tag. The resulting recombinant plasmids were verified by DNA sequencing, followed by transformation into the *E. coli* expression strain BL21 (DE3). *E. coli* samples before and after the transformation were prepared for Western blot assays.

### FLJ33706 antiserum, brain sample preparation, and Western blot analyses

A 17-amino-acid peptide with sequence CTSKAQRVHPQPSHQRQ that corresponded to the unique residuals 68–83 of FLJ33706 putative protein was synthesized (cystine was added to conjugate to keyhole limpet hemocyanin) and used to immunize rabbits (Genemed Synthesis, Inc., San Antonia, TX, USA). The peptide sequence is highly antigenic and lacks detectable homologues in any mammalian genomes based on BLASTP. The FLJ33706-specific anti-serum was produced in a favourable animal after initial and boosting immunizations. Protein levels were quantitated using Bradford assays and 50 µg protein aliquots of supernatant were electrophoresed using 10% SDS-polyacrylamide gels and Western blot analysis was performed as described previously [25]. FLJ33706 anti-serum that was diluted 1∶5000 and the pre-immune serum that was diluted with 1∶5000 were used to replace anti-FLJ33706 serum. The synthetic peptide (100 µg/ml) was incubated with primary antiserum that had been pre-absorbed 2 h at room temperature for the competition assay [25]. Western blot assays with *E. coli* expressed FLJ33706 recombination protein (with His-Tag) were also introduced to evaluate the specificity of FLJ33706 antibody, in which anti-FLJ33706 and anti-His tag was diluted at 1∶5000 and 1∶500, respectively.

### Immunohistochemistry

Immunohistochemistry study of FLJ33706 in human brain cortex was performed as previously described [42]. Antiserum of FLJ33706 is produced as mentioned above (1∶400), and antibody against beta-tubulin III was ordered from Sigma (1∶200).

## Supporting Information

Table S1 Quantification of *FLJ33706* mRNA levels
(0.07 MB DOC)

Table S2 Population distribution of 90 individuals used in population genetics study
(0.03 MB DOC)

Figure S1 The DNA segment where *FLJ33706* is located emerged in the eutherian mammals. For the chromsome region of *FLJ33706*, the 44-way vertebrate syntenic genome-alignment tracks of the UCSC browser were shown. The alignments suggest that the DNA segment where *FLJ33706* is located emerged in the eutherian mammals, since it is complete absent from all outgroups ranging from marsupials to lamprey.
(0.43 MB TIF)

Figure S2 Multiple sequencing reads support that chimpanzee share the ancestral status of the disablers of FLJ33706 proper open reading frame. The figure showed that a stop codon (TAG) in chimpanzee is supported by six reads, thus unlikely to be caused by sequencing errors.
(0.04 MB TIF)

Figure S3 Sliding window analysis of nucleotide diversity. The boxes above the bottom line mark the location of exons (Exon 2, 3 and 4) by scale. ‘ATG’ and ‘TAG’ indicate the start codon and stop codon respectively. The yellow boxes below the bottom line show the repeat elements annotated by UCSC genome browser. As the figure shows, all four notable polymorphism peaks concur with non-CDS regions such as introns or 3′ UTR. If we consider that repeat elements might help to facilitate recombination and thus increase pi, the constraint of CDS is even more pronounced since almost the whole first coding exon is covered by a repeat element.
(0.09 MB TIF)

Figure S4 Significantly higher *FLJ33706* mRNA expression levels were detected in human AD brains. The transcript expression level of *FLJ33706* in 20 Alzheimer's disease (AD) brains and 18 normal brains were tested. The transcript expression level of *FLJ33706* in AD brains is significantly elevated in Alzheimer's disease (AD) brains (Mann Whitney Test p = 0.0273). CNT: normal brain tissues; AD: Alzheimer's disease brain tissues.
(0.13 MB TIF)

Figure S5 Introns 2 and 3 of *FLJ33706* are enriched with fast-evolving nucleotides. We downloaded phyloP conservation score based on placental mammal genome alignment from UCSC table browser and then made a smoothed plot with Geneplotter package in R. Positive score means constraint, while negative score indicates positive selection. The pink box marks a fast-evolving peak, which concurs with the second and third introns.
(0.69 MB TIF)
