# Supplementary material for: A Human-Specific De Novo Protein-Coding Gene Associated with Human Brain Functions
Source: PLoS Comput Biol. 2010 Mar 26;6(3):e1000734. doi: 10.1371/journal.pcbi.1000734 (PMC2845654; doi:10.1371/journal.pcbi.1000734)
Supplement: Table S1 — Quantification of FLJ33706 mRNA levels (0.07 MB DOC) [file pcbi.1000734.s001.doc]

**Supplementary Table S1: quantification of *FLJ33706* mRNA levels**

*FLJ33706* mRNA levels were measured in eight human peripheral tissues and eight human brain regions using TaqMan-based Real-Time PCR system. Relative quantity (ddCt) was calculated using expression means of human leukocyte as references.

| **TISSUES** | **FLJ33706**  **Ct1** | **FLJ33706**  **Ct2** | **FLJ33706**  **Ct3** | **FLJ33706**  **Ctavg** | **GAPDH**  **Ct1** | **GAPDH**  **Ct2** | **GAPDH**  **Ct3** | **GAPDH**  **Ctavg** | **dCt** | **ddCt** | **Fold** |
| --- | --- | --- | --- | --- | --- | --- | --- | --- | --- | --- | --- |
| FLJ_Intestine | 33.97 | 35.74 | 34.61 | 34.78 | 19.52 | 19.55 | 19.55 | 19.54 | 15.24 | 2.0 | 0.2 |
| FLJ_Kidney | 34.70 | 34.72 | 33.81 | 34.41 | 19.30 | 19.28 | 19.27 | 19.28 | 15.13 | 1.9 | 0.3 |
| FLJ_Leukocyte | 32.96 | 32.11 | 32.42 | 32.50 | 19.25 | 19.31 | 19.25 | 19.27 | 13.23 | 0.0 | 1.0 |
| FLJ_Liver | NS* | NS | NS | NS | 20.51 | 20.48 | 20.47 | 20.49 | NS | NS | 0.0 |
| FLJ_Spleen | 34.09 | 36.07 | 35.74 | 35.30 | 21.81 | 21.69 | 21.65 | 21.72 | 13.58 | 0.4 | 0.8 |
| FLJ_Testis | 32.21 | 32.58 | 32.32 | 32.37 | 19.51 | 19.95 | 19.58 | 19.68 | 12.69 | -0.5 | 1.5 |
| FLJ_Heart | 33.59 | 33.52 | 34.40 | 33.84 | 17.91 | 17.87 | 17.82 | 17.87 | 15.97 | 2.7 | 0.1 |
| FLJ_Lung | NS | NS | NS | NS | 19.22 | 19.72 | 19.66 | 19.53 | NS | NS | 0.0 |
| FLJ_Amygdala | 33.13 | 32.95 | 32.65 | 32.91 | 21.69 | 21.65 | 21.64 | 21.66 | 11.25 | -2.0 | 3.9 |
| FLJ_Caudate | 31.28 | 31.45 | 31.27 | 31.33 | 19.36 | 19.28 | 19.38 | 19.34 | 11.99 | -1.2 | 2.4 |
| FLJ_Cerebellum | 30.39 | 30.53 | 29.89 | 30.27 | 18.71 | 18.52 | 18.52 | 18.58 | 11.69 | -1.5 | 2.9 |
| FLJ_Cortex | 30.37 | 29.94 | 30.01 | 30.11 | 18.57 | 18.57 | 18.52 | 18.55 | 11.56 | -1.7 | 3.2 |
| FLJ_Hippocampus | 30.97 | 30.96 | 30.51 | 30.81 | 19.26 | 19.05 | 18.99 | 19.10 | 11.71 | -1.5 | 2.9 |
| FLJ_Nuclear Accumbens | 29.79 | 29.50 | 29.39 | 29.56 | 18.83 | 18.52 | 18.50 | 18.62 | 10.95 | -2.3 | 4.9 |
| FLJ_Puteman | 31.92 | 31.67 | 31.70 | 31.76 | 20.73 | 20.67 | NS | 20.70 | 11.06 | -2.2 | 4.5 |
| FLJ_Substantial Nigra | 30.09 | 30.48 | 30.05 | 30.21 | 18.46 | 18.45 | 18.40 | 18.44 | 11.77 | -1.5 | 2.7 |
| BDNF1_CTX | 32.68 | 32.79 | 32.50 | 32.66 | 19.99 | 20.46 | 19.73 | 20.06 | 12.60 | -0.6 | 1.6 |
| BDNF4_CTX | 29.58 | 29.59 | 29.43 | 29.53 | 19.99 | 20.46 | 19.73 | 20.06 | 9.47 | -3.8 | 13.5 |

* NS means no expression signal detected.
