# Supplementary material for: A Human-Specific De Novo Protein-Coding Gene Associated with Human Brain Functions
Source: PLoS Comput Biol. 2010 Mar 26;6(3):e1000734. doi: 10.1371/journal.pcbi.1000734 (PMC2845654; doi:10.1371/journal.pcbi.1000734)
Supplement: Table S2 — Population distribution of 90 individuals used in population genetics study (0.03 MB DOC) [file pcbi.1000734.s002.doc]

**Supplementary Table S2: population distribution of 90 individuals used in population genetics study**

| **Populations** | **Individuals** |
| --- | --- |
| AFRICAN AMERICAN | NA17031, NA17032, NA17033, NA17034, NA17035, NA17036, NA17037, NA17038, NA17039, NA17040 |
| BIAKA PYGMY POPULATION | NA10469, NA10470, NA10471, NA10472, NA10473, NA10492, NA10493, NA10494, NA10495, NA10496 |
| AFRICAN SOUTH OF THE SAHARA | NA17341, NA17342, NA17343, NA17344, NA17345, NA17346, NA17347, NA17348, NA17349 |
| CHINESE | NA17014, NA17015, NA17016, NA17017, NA17018, NA17019, NA17020, NA16654, NA16688, NA16689 |
| RUSSIAN | NA13820, NA13838, NA13849, NA13852, NA13876, NA13877, NA13911, NA13912, NA13913, NA13914 |
| INDO PAKIST ANI | NA17024, NA17026, NA17027, NA17029, NA17030 |
| JAPANESE | NA17051, NA17052, NA17053, NA17054, NA17055, NA17056, NA17057, NA17058, NA17059, NA17060 |
| SOUTH AMERICA | NA17311, NA17312, NA17313, NA17314, NA17315, NA17316, NA17317, NA17318, NA17319, NA17320 |
| ASKENAZI JEWISH | NA17360, NA17361, NA17362, NA17363, NA17364, NA17365, NA17366, NA17367, NA17368, |
| AFRICANS NORTH OF THE SAHARA | NA17378, NA17380, NA17381, NA17384, NA17392, NA17394, NA17396 |
