# Supplementary figures and images for: A Human-Specific De Novo Protein-Coding Gene Associated with Human Brain Functions
Source: PLoS Comput Biol. 2010 Mar 26;6(3):e1000734. doi: 10.1371/journal.pcbi.1000734 (PMC2845654; doi:10.1371/journal.pcbi.1000734)

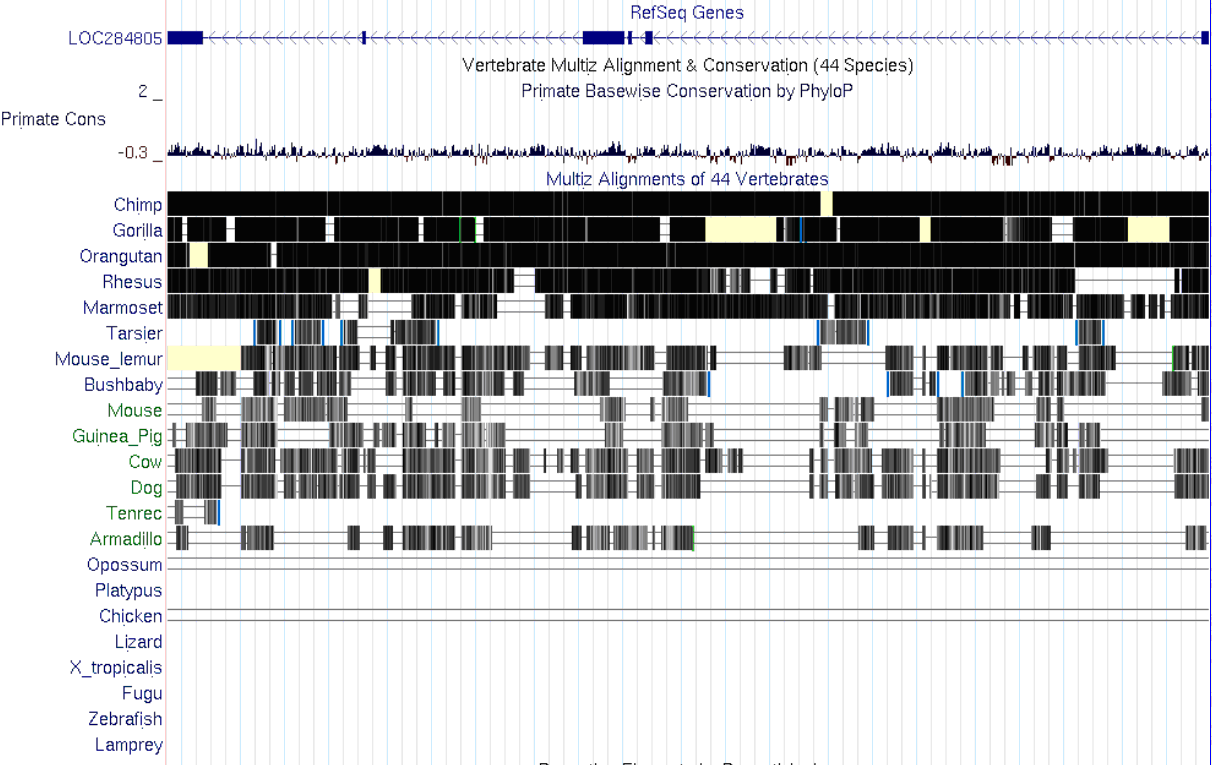

Supplement: Figure S1 — The DNA segment where FLJ33706 is located emerged in the eutherian mammals. For the chromsome region of FLJ33706, the 44-way vertebrate syntenic genome-alignment tracks of the UCSC browser were shown. The alignments suggest that the DNA segment where FLJ33706 is located emerged in the eutherian mammals, since it is complete absent from all outgroups ranging from marsupials to lamprey. (0.43 MB TIF) [file pcbi.1000734.s003.tif]

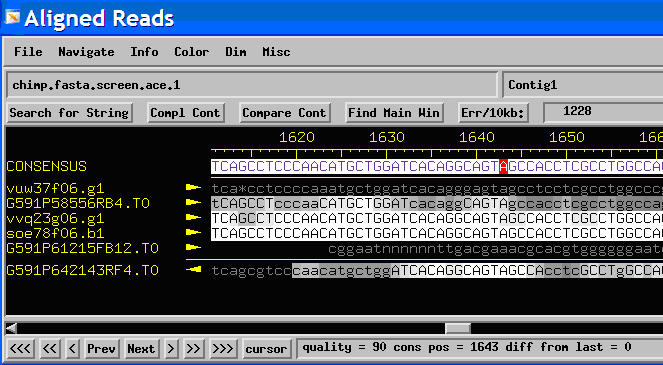

Supplement: Figure S2 — Multiple sequencing reads support that chimpanzee share the ancestral status of the disablers of FLJ33706 proper open reading frame. The figure showed that a stop codon (TAG) in chimpanzee is supported by six reads, thus unlikely to be caused by sequencing errors. (0.04 MB TIF) [file pcbi.1000734.s004.tif]

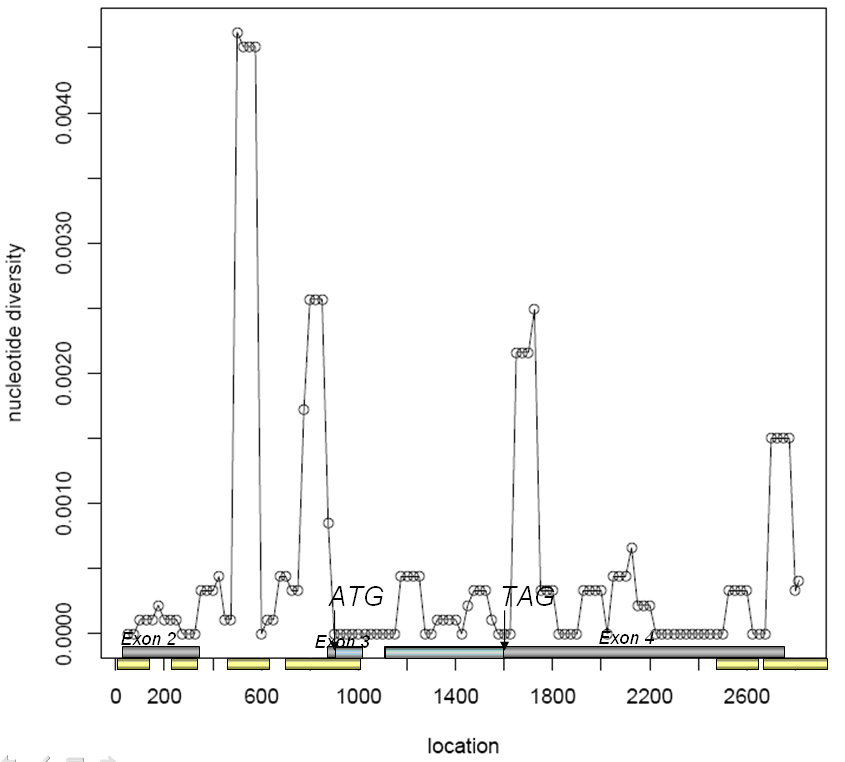

Supplement: Figure S3 — Sliding window analysis of nucleotide diversity. The boxes above the bottom line mark the location of exons (Exon 2, 3 and 4) by scale. ‘ATG’ and ‘TAG’ indicate the start codon and stop codon respectively. The yellow boxes below the bottom line show the repeat elements annotated by UCSC genome browser. As the figure shows, all four notable polymorphism peaks concur with non-CDS regions such as introns or 3′ UTR. If we consider that repeat elements might help to facilitate recombination and thus increase pi, the constraint of CDS is even more pronounced since almost the whole first coding exon is covered by a repeat element. (0.09 MB TIF) [file pcbi.1000734.s005.tif]

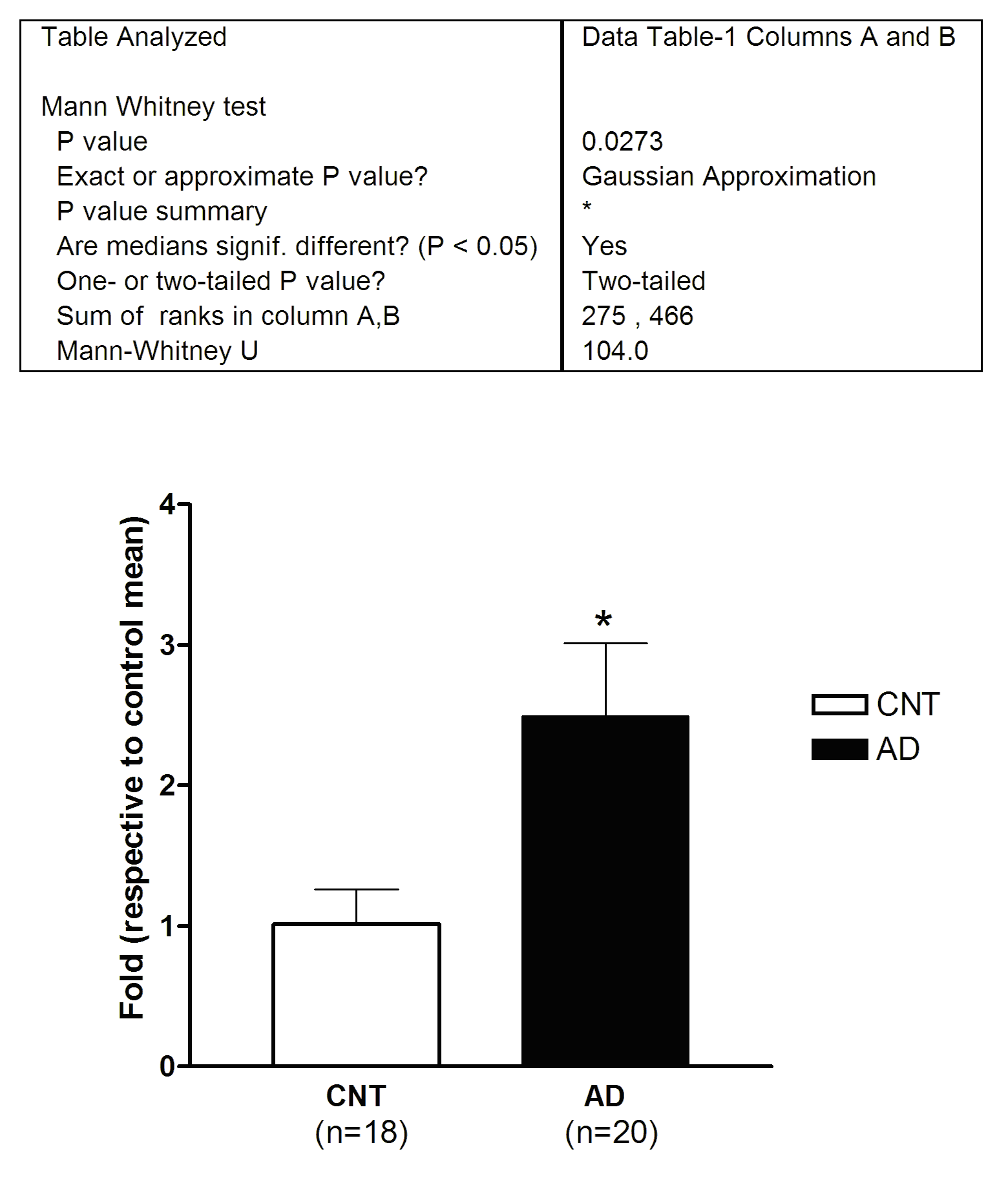

Supplement: Figure S4 — Significantly higher FLJ33706 mRNA expression levels were detected in human AD brains. The transcript expression level of FLJ33706 in 20 Alzheimer's disease (AD) brains and 18 normal brains were tested. The transcript expression level of FLJ33706 in AD brains is significantly elevated in Alzheimer's disease (AD) brains (Mann Whitney Test p = 0.0273). CNT: normal brain tissues; AD: Alzheimer's disease brain tissues. (0.13 MB TIF) [file pcbi.1000734.s006.tif]

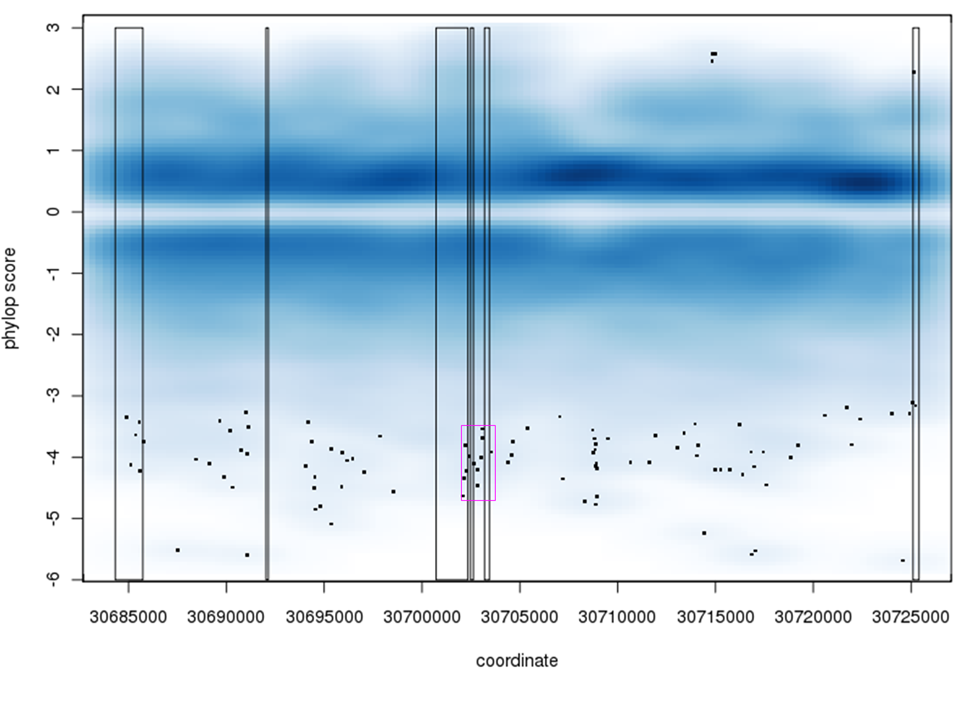

Supplement: Figure S5 — Introns 2 and 3 of FLJ33706 are enriched with fast-evolving nucleotides. We downloaded phyloP conservation score based on placental mammal genome alignment from UCSC table browser and then made a smoothed plot with Geneplotter package in R. Positive score means constraint, while negative score indicates positive selection. The pink box marks a fast-evolving peak, which concurs with the second and third introns. (0.69 MB TIF) [file pcbi.1000734.s007.tif]
